# Insight into the Earthquake Risk Information Seeking Behavior of the Victims: Evidence from Songyuan, China

**DOI:** 10.3390/ijerph14030267

**Published:** 2017-03-07

**Authors:** Shasha Li, Guofang Zhai, Shutian Zhou, Chenjing Fan, Yunqing Wu, Chongqiang Ren

**Affiliations:** 1 School of Geographic and Oceanographic Sciences, Nanjing University, Nanjing 210046, China; lisa851214@163.com (S.L.); c1f5_e9aa@163.com (C.F.); rcq518@163.com (C.R.); 2 School of Architecture and Urban Planning, Nanjing University, Nanjing 210093, China; 15996541020@163.com; 3 College of Geomatics Engineering, Nanjing Tech University, Nanjing 210009, China; njwuyunqing@163.com; 4 College of Economics, Northwest University for Nationalities, Lanzhou 730030, China

**Keywords:** earthquake risk, information seeking behavior, information need, risk communication, structural equation model, China

## Abstract

Efficient risk communication is a vital way to reduce the vulnerability of individuals when facing emergency risks, especially regarding earthquakes. Efficient risk communication aims at improving the supply of risk information and fulfilling the need for risk information by individuals. Therefore, an investigation into individual-level information seeking behavior within earthquake risk contexts is very important for improved earthquake risk communication. However, at present there are very few studies that have explored the behavior of individuals seeking earthquake risk information. Under the guidance of the Risk Information Seeking and Processing model as well as relevant practical findings using the structural equation model, this study attempts to explore the main determinants of an individual’s earthquake risk information seeking behavior, and to validate the mediator effect of information need during the seeking process. A questionnaire-based survey of 918 valid respondents in Songyuan, China, who had been hit by a small earthquake swarm, was used to provide practical evidence for this study. Results indicated that information need played a noteworthy role in the earthquake risk information seeking process, and was detected both as an immediate predictor and as a mediator. Informational subjective norms drive the seeking behavior on earthquake risk information through both direct and indirect approaches. Perceived information gathering capacity, negative affective responses and risk perception have an indirect effect on earthquake risk information seeking behavior via information need. The implications for theory and practice regarding risk communication are discussed and concluded.

## 1. Introduction

Risk information has become permanently available and accessible with the development of modern communication media and the popularity of the Internet [1,2]. Nonetheless, only a limited number of studies have aimed to explain what prompts individuals to engage in and seek personally relevant risk information. Due to this gap in the literature, a new research direction focusing on individual-level risk information seeking behavior has been developed in risk communication research [2,3,4,5], especially related to public health risks. Some models have been proposed to explain the behavior of individuals seeking risk information. The most oft-cited model is the Risk Information Seeking and Processing model (RISP) [4]. This model attracts great attention due to the proposed predictors of individual-level information behavior and the universal applicability of risk situations. Based on the RISP model, the Framework for Risk Information and Seeking (FRIS) [1] and the Planned Risk Information Seeking Model (PRISM) [6] were developed.

The RISP model and its subsequent iterations have been applied to environmental and health risk issues related to the environment. For instance, the consumption risk of contaminated fish [7,8,9]; municipal drinking water polluted by chemicals and organisms risk [7,8,9,10,11]; use of renewable energy sources and ecological security [8,9,12]; hazardous industrial risk and hazard waste transportation [2,13]; flood risks [3,14,15]; climate change [16]; and global warming [17]. In these studies, the RISP model has been constantly improved; however, the role of information need in the seeking process has not been fully explored. Furthermore, to date, hardly any studies have tested the RISP model in the seismological fields.

Efforts to understand what influences the behavior of individuals seeking earthquake risk information is important in earthquake risk management. Earthquakes are one of the most unpredictable and destructive natural disasters and often cause heavy casualties and economic losses. The reduction of an individual’s vulnerability can contribute to the decrease in huge losses of economy and casualties caused by earthquakes. So, the question of how to effectively reduce the vulnerability of individuals has become a ubiquitous problem that challenges earthquake sciences. One crucial measure is to improve the public’s capability in emergency response and adaptation in earthquake hazard prone areas. This requires individuals to gain insight into the knowledge and information on earthquake risk. Effective risk information helps an individual to judge earthquake risk situations, so that they can make accurate decisions and take quick actions regarding mitigation measures and adjustments [1,14,18]. Towards this end, efficient earthquake risk communication efforts are indispensable to satisfy the needs of specific earthquake risk information [18] and to improve earthquake risk information supply to the public.

Based on this background and the combination of the adapted RISP model and related work application in earthquake situations, this current study effort attempted to realize two research objectives. The first research objective was to map the main determinants of earthquake risk information seeking behavior. The relationships among these predictors were also analyzed through this process. The second research objective was to validate the mediating effect of information need between earthquake risk information seeking behavior and the predictors, to fully explore the role of the information need. It is noteworthy that the target group are the direct victims who had just suffered from a small earthquake swarm in China, and is different from previous research which targeted potential victims of a certain risk.

This study is presented in six parts. Following the introduction, Section 2 reviews information-seeking models and key model components through the theoretical lens, then puts forward research hypotheses and a conceptual model of earthquake risk information-seeking behavior. Section 3 introduces the study area, the use of the questionnaire-based survey and data analysis techniques. The research findings are highlighted and discussed in Section 4 and Section 5. The last section presents our conclusion and discusses the limitations, future studies and implications of this research.

## 2. Theoretical Background and Research Hypotheses

### 2.1. Theoretical Lens

In this Section, we first reviewed the literature on Information Seeking Models [1,4,6] to obtain the determinants and their relationships with information seeking behavior. Next, a detailed introduction was given to the key components in our model.

#### 2.1.1. A Literature Review on Information Seeking Models

The RISP model was proposed by Griffin et al. [4] to guide empirical research [19], and aimed to provide a theoretical framework for mapping the potential predictors of information seeking and processing within risk contexts. Some of the concepts in the model were derived from the Heuristic-Systematic Processing Model [20] and the Theory of Planned Behavior (TPB) [21,22,23,24]; and others from the risk research [25,26,27] and mass media research disciplines [28,29,30,31].

Early in the end of 20th century, Griffin et al. [4] proposed information insufficiency, perceived information-gathering capacity and relevant channel beliefs, as the three direct determinants of information seeking behavior. Furthermore, some indirect predictors, for instance, individual characteristics; perceived hazard characteristics; informational subjective norms; and affective responses were also involved within this model. The key concept of the model was information insufficiency. Griffin et al. [3] had adjusted affective responses (anger, for example) and informational subjective norms as the direct predictors to information seeking behavior in 2008, and found that information insufficiency; perceived information gathering capacity; informational subjective norms; and affective responses had positive relationships with information seeking behavior.

Based on the original RISP model, a theoretical framework called FRIS was developed by Ter Huurne [1]. The FRIS added social-psychological variables—institutional trust, engagement and self-efficacy—related to risk. Empirical results [2] illustrated that information needs, risk perception, and current knowledge affected the information seeking intention directly. Ter Huurne et al. [13] proposed two new direct motivators of information seeking, which were informational subjective norms and affective responses. The new relationship between perceived information gathering capacity and current knowledge was also explored.

The focus of Kahlor’s effort was an augmented version of the RISP model, with two additional concepts—attitude and intention—from the TPB [17]. This augmented model was improved by the subsequent addition of five new relationships and was referred to as the PRISM [6]. This research proved that seeking-related subjective norms; affective risk responses; attitude toward seeking; and perceived seeking control were the direct predictors for information seeking intent (akin to information seeking behavior). However, this model failed to illustrate the vital correlation between knowledge insufficiency and seeking intent.

Kellens et al. [14] built a model upon the above information seeking models to explore the main determinants of information seeking behavior. The results showed that risk perception, response efficacy, and perceived hazard knowledge had positive correlations with information seeking behavior. Furthermore, Kellens et al. [14] paid considerable attention to information need as the mediator. However, since the direct effect of information need on information seeking behavior failed to be proven, this resulted in the failure of information need as a mediating role.

#### 2.1.2. Key Model Components

A. Information seeking behavior (Behavior)

There is no unified definition regarding information seeking behavior. Wilson [32] identified information seeking behavior as “the purposive seeking for information as a consequence of a need to satisfy some goal”. Johnson [33] defined it as “the purposive acquisition of information from selected information carriers”. Krikelas [16] considered information seeking behavior to be “any activity of an individual that is undertaken to identify a message that satisfies a perceived need”. Machionini [34] defined information seeking behavior as “a process in which humans purposefully engage in order to change their state of knowledge”. Griffin et al. [4] defined it as “the effort to acquire information in response to a need or perceived gap in one’s knowledge”.

In conclusion, information seeking behavior can be described as deliberate behavior focused on the acquisition of information. It can vary in breadth and depth, and is goal driven to satisfy a perceived need for information. When there is no need, the individual will withdraw from the seeking process. Information seeking behavior is the main dependent variable in this study.

B. Information need (Need)

Information insufficiency (which is akin to information need) is the core concept of the RISP model. It manifests the perceived gap between the existing risk knowledge and needful risk knowledge to decide how an individual behaves when faced with risky situations. As seen in the above definitions of information seeking behavior, information need is the central driving factor for information seeking. That is, when an individual feels unsatisfied with their current information status and desires more additional information about the risk, they will put more effort into seeking out risk information. Some former studies [2,3,8,13,16,17,19] have proven this; however, there are also other studies [6,14] that have failed to confirm this outcome.

C. Current risk knowledge (Knowledge)

In the RISP model, as part of information insufficiency, current risk knowledge is not correlated with any other model concepts [6]. However, other researchers have suggested that current risk knowledge is a truly independent variable [2,6,8,13,14,17], a stance also taken in this paper.

Acquiring knowledge is one of the most basic drivers of information seeking for individuals [1], especially if the knowledge is relevant and important to themselves, such as risk knowledge about their environment. As knowledge accumulates, if an individual feels that they have adequate risk knowledge, they may cease risk information seeking behavior. However, if their knowledge is perceived to be inadequate, the individual will seek risk information more actively. In other words, lower level of existing risk knowledge leads to a higher level of information need [14] and thus more effortful information seeking [2,8,13,14].

D. Perceived information gathering capacity (Capacity)

According to Griffin et al. [3,4], when an individual performed the information seeking steps to access and understand more information, such as information about risk in one’s neighborhood and response to that risk, it requires a perceived ability, called the perceived information gathering capacity, as a pattern of an individual’s sense of self-efficacy [35,36] or perceived behavioral control [21,22]. Previous studies have found that current risk knowledge affected perceived information gathering capacity positively [3,6,8,13]. Simultaneously, the RISP model and related research argued that perceived information gathering capacity was an influential factor that drove one’s information seeking behavior [3,6,8,13,14,19]. In this study, whether the perceived information gathering capacity positively affected information need was explored.

E. Informational subjective norms (Norm)

Informational subjective norms are socio-psychological variables derived from the theory of Planned Behavior [37], meaning “the perceived social pressure to perform or not to perform a certain behavior” [21]. When basic information about a specific risk issue is acknowledged by one’s family members and friends, the individual will feel pressure or expectation to keep on top of information regarding this risk. They will therefore have a sense of greater information need and thus seek risk information more actively. Previous empirical research has found that informational subjective norms positively affected one’s current risk knowledge [6,13,16,19], information need [3,6,7,8,9,11,13,17], and also risk information seeking behavior [3,6,7,8,9,11,13,17,19,26].

F. Negative affective responses (Affect)

Affective responses refer to emotional reactions, including negative and positive feelings, which seem to play a role in public risk judgments. When individuals are facing risk, most of their feelings are likely to be negative in nature. Negative affective responses lead to the awareness of a lack of risk knowledge [13,37], and subsequently, greater perceived information need [3,6,7,8,9,11,16,17], which ultimately prompts more active seeking behavior for risk information [3,4,6,8,13,16,17,19]. Many previous studies have also emphasized the negative affect; however, positive affective responses were also explored by Yang and Kahlor [38], and found that positive emotion could also be a direct determinant of risk information seeking behavior [39].

G. Risk perception (Perception)

Risk perception plays an important role in improving risk communication. It represents the public’s subjective judgments of the probability (or vulnerability) of a hazard and the severity of its potential consequences. An individual’s perception of a certain risk may affect the behavior with which they respond to that risk. Several studies support the statement that the higher the probability (or vulnerability) and severity a risk is perceived to be, the more negative affect may emerge [3,7,13,16,17], which leads to more perceived information need [2,14,40], thus more information seeking behavior [2,14,40].

### 2.2. Research Hypotheses

**Figure 1:**
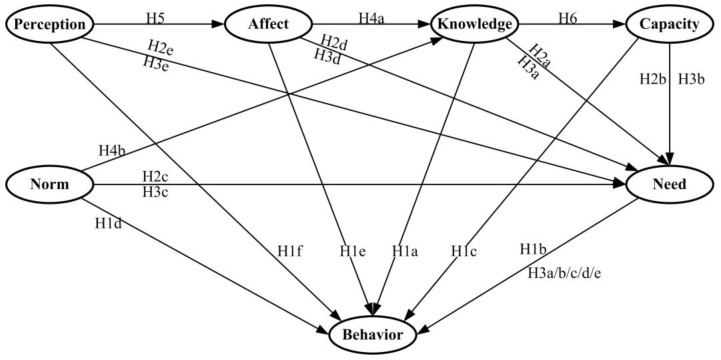
Hypothesized relationships among determinants of information seeking behavior. Behavior: Information seeking behavior; Knowledge: Current risk knowledge; Need: Information need; Capacity: Perceived information gathering capacity; Norm: Informational subjective norms; Affect: Negative affective responses; Perception: Risk perception.

Based upon the theoretical assumptions by the aforementioned literature, the research hypotheses (H) describe the earthquake risk information seeking behavior’s determinants, the relationships between these determinants, and the mediator role of information need, as presented below (see Figure 1):
H1a/b/c: current risk knowledge (a); information need (b); and perceived information gathering capacity (c) are positively related to earthquake risk information seeking behavior;H1d/e/f:informational subjective norms (d); negative affective responses (e); and risk perception (f) are positively related to earthquake risk information seeking behavior;H2a:current risk knowledge (a) is negatively associated with information need;H2b/c/d/e:perceived information gathering capacity (b); informational subjective norms (c); negative affective responses (d) and risk perception (e) are positively associated with information need;H3a/b/c/d/e:information need mediates the H1a effect (a); H1c effect (b); H1d effect (c); H1e effect (d) and H1f effect (e);H4a/b:negative affective responses (a) and informational subjective norms (b) have a positive effect on current risk knowledge;H5:risk perception has a positive effect on negative affective responses;H6:current risk knowledge has a positive influence on perceived information gathering capacity.

## 3. Materials and Methods

### 3.1. Study Area

**Figure 2:**
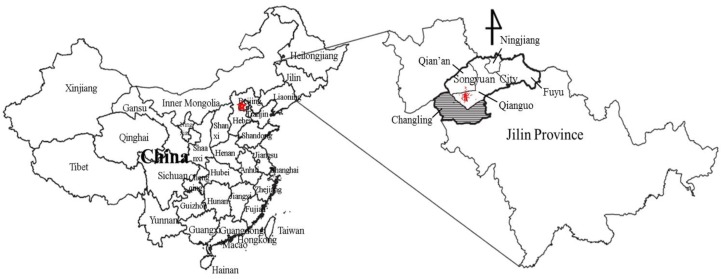
Location of study area. The red spot in Jilin Province indicates the epicenters of the earthquake swarm. The shaded part is the survey area.

The area of interest in this study is Songyuan (Figure 2), a city in the midwest of Jilin province, China, located at 123°6′–126°11′E and 43°59′–45°32′N. Songyuan is the only VIII degree high intensive seismic region in Jilin Province, and is determined as one of the key monitoring defensive regions of China. There are three tectonic fractures in Songyuan: the northeast trending Fuyu-Zhaodong fault; the north-west trending Second Songhua River fault; and the north-north trending Nen River fault. Songyuan City includes Changling County; Qianguo County; Qian’an County; Fuyu County-level City; and Ningjiang District.

From 31 October 2013, to 3 March 2014, 1017 earthquakes, including five strong earthquakes with the magnitude 5.0 or larger occurred in Songyuan City and caused severe damage. Thirteen people were injured [41]; and more than 57,000 houses were damaged in Qianguo County, Qian’an County, and Changling County, including 16,000 houses severely destroyed; 40,000 houses slightly damaged and nearly 310 houses collapsed [42]. The economic loss is up to ¥20 billion [42]. The worst-hit areas were Chaganhua Town in Qianguo County and Anzi Town in Qian’an County (area of around epicenter, red zone in Figure 2). The earthquakes occurred in winter where the extreme minimum temperature was −36.1 °C in the affected areas. As the damaged houses were unable to be repaired and rebuilt in the winter, the victims had to join relatives and friends to find accommodation or rent houses in other areas to get through the winter, which meant that all the people in the two towns left the area. Thus, this study chose Changling County as the survey area, given that it was the nearest to the epicenter and therefore more representative.

### 3.2. Questionnaire Design 

The anonymous questionnaire in Mandarin consisted of two sections. The first section collected the information of the socioeconomic and housing characteristics of the respondents, such as gender, age; education; income; housing ownership; housing type; and housing structure. The second section asked questions on information seeking behavior and its possible determinants. Some items (such as information seeking behavior [2,3]; information need [13]; perceived information gathering capacity [3]; informational subjective norms [1,3,38]; negative affective responses [2,13,38]; risk perception [43]) were measured on the basis of previous empirical studies, and current risk knowledge which was newly developed. The exact formulation of the items is presented in Table 1. All 28 items were measured by a five-point Likert-type scale ranging from 1 to 5, with higher scores indicating higher agreement with the statement.

### 3.3. Implementation

The data were collected in Changling County in Songyuan City, China in March 2014. Working through the Changling County Education Bureau, six representative schools (No. 1 Primary School; No. 2 Primary School; No. 3 Primary School; No. 4 Primary School; No. 1 Junior High School; and No. 3 Senior High School) were selected for participation. The six schools were all part of the school district system, leading to the group distribution of the surveys, therefore achieving wide coverage. With permission from the selected local schools, school students received the questionnaires as a form of homework and delivered it to one of their guardians. A total of 1000 questionnaires were sent out, and 973 were completed with a response rate of 97.3%. There were two possible reasons for this high response rate: one was the recent earthquake swarm experience, so the topic of this survey related to the parents’ own daily lives; and the second was due to the respect paid by Chinese parents to the teachers’ authority (who usually follow the teachers’ requests) [44].

### 3.4. Analysis Techniques

The statistical analysis was carried out in four parts. Firstly, a simple frequency analysis was used to describe the socioeconomic and housing characteristics of the respondents. Secondly, the 28 original items were estimated with Cronbach’s α coefficients. This consisted of an exploratory factor analysis (EFA) was performed to extract dimensions before using the Kaiser-Meyer-Olkin (KMO) and Bartlett’s test of sphericity to test if the results of exploratory factor analysis were appropriate. The Cronbach’s α coefficients and the item-total correlations were also calculated to evaluate the reliability and validity. Thirdly, a confirmatory factor analysis (CFA) was implemented to assess the stability of the relationship between the structural factors and the measurement items from the aforementioned EFA, and to identify the existence of cross validity. Finally, a structural equation modelling (SEM) was used to test the proposed conceptual model of the impacts on earthquake risk information seeking behavior.

## 4. Results

### 4.1. Socioeconomic and Housing Characteristics of Respondents

Among the 973 returned questionnaires, 918 questionnaires offered valid responses. Males were slightly overrepresented (52.1%). Approximately 90% of survey participants were between the ages of 31–50. Approximately half of them had a high school degree or above, and over 90% of respondents earned less than ¥100,000 per family per year. More than 80% owned their own house, with bungalows (43.9%) and multilayer buildings (47.7%) as the main housing styles, and masonry-concrete structures (52.4%) and reinforced-concrete structures (44%) as the main housing structures.

### 4.2. Exploratory Factor Analysis and Reliability Analysis

The questionnaire of earthquake risk information seeking behavior contained 28 original items (four of them were eliminated via reliability analysis). The EFA was undertaken using the remaining 24 items to extract dimensions. According to the results of the KMO and Bartlett’s test of sphericity, the KMO of 0.89 was greater than 0.80, with p < 0.001. This indicated that this group of data was suitable for the EFA [45]. The total variance explained was 67.08%, above the common standard of 60% in the sphere of the social sciences [46]. Furthermore, all 24 remaining items had a factor loading greater than 0.5, which corresponded to seven dimensions (Table 1). 

According to the reliability test, the Cronbach’s α values ranged from 0.64–0.88 (Table 1), which were higher than the threshold value of 0.60 [47]. In accordance with the validity test, the item-total correlations were between 0.36–0.59, greater than 0.3 [48]. Therefore, the data’s internal consistency was good and completely valid for further analyses with a perfect internal validity as had the factor analysis data. This certified that the EFA was reasonable and acceptable. Hence, we labeled the seven constructs as information seeking behavior (Behavior); current risk knowledge (Knowledge); information need (Need); informational subjective norms (Norm); perceived information gathering capacity (Capacity); negative affective responses (Affect); and risk perception (Perception) (Table 1).

### 4.3. Confirmatory Factor Analysis

Using the maximum likelihood estimation, the CFA with a first-order and one-dimension was computed to measure the seven constructs. As shown in Table 2, inter-item correlations (construct loadings) were extremely significant in statistics (p < 0.001, all above the threshold value of 0.50 [49]), which indicated that the latent variables could be strongly explained by measured variables. The composite reliability values (CR) ranged from 0.61–0.93, greater than the critical limit (0.60) [50]. The average variance extracted (AVE) values were between 0.35–0.73. The AVE values of Knowledge (0.43) and Affect (0.47) were near the threshold value of 0.50 [51], but Behavior (0.35) and Capacity (0.37) were lower, suggesting that the average explanatory power of each item was insufficient to represent the latent constructs of both Behavior and Capacity; while the other latent constructs had good reliability.

The results of the fit indices are shown in Table 2. Model fit was adequate with a root mean square error of approximation (RMSEA) of 0.05 (<0.08); a λ^2^/degree of freedom (df) ratio of 3.48 (<5); a comparative fit index (CFI) of 0.93; a goodness of fit index (GFI) of 0.93; and an adjust goodness of fit index (AGFI) of 0.91. Nearly all indices were within the limits of the threshold values recommended by previous studies [52,53,54,55], indicating that this conceptual model would be statistically acceptable on the basis of the good fit test.

### 4.4. Structural Model and Hypothesis Testing

**Figure 3:**
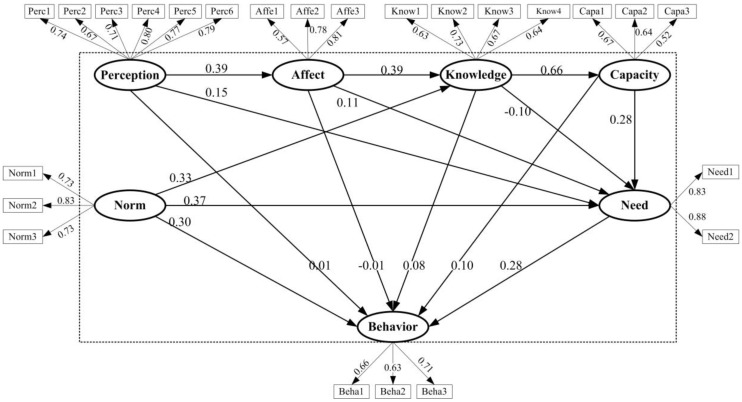
Results of the structural equation model. Behavior: Information seeking behavior; Knowledge: Current risk knowledge; Need: Information need; Capacity: Perceived information gathering capacity; Norm: Informational subjective norms; Affect: Negative affective responses; Perception: Risk perception. Solid lines: Significant at *p* < 0.01; dotted lines: Insignificant.

After the CFA, the structural model was assessed using model fit measurements. Table 3 shows the results of the model estimation; fitness; and evaluation for the hypothetical models above. The model fit was at an acceptable level (λ^2^/df = 4.42, RMSEA = 0.06, GFI = 0.91, AGFI = 0.89). The results of the causal relationships and correlations of each path can be seen in Table 4 and Figure 3.

The first set of hypotheses explored the direct determinants of earthquake risk information seeking behavior (ERISB). H1b hypothesized that information need was positively related with ERISB, and that path coefficient was 0.28 (*p* < 0.001), in support of H1b. Similarly, informational subjective norms had a positive relationship with ERISB (β1d = 0.30, *p* < 0.001), also in support of H1d. Furthermore, the path coefficients between current risk knowledge to ERISB (β1a = 0.08, non-significant, n.s.); perceived information gathering capacity to ERISB (β1c = 0.10, n.s.); negative affective responses to ERISB (β1e = −0.01, n.s.); and risk perception to ERISB (β1f = 0.01, n.s.) were statistically insignificant, failing to support H1a, H1c, H1e and H1f. Thus, current risk knowledge; perceived information gathering capacity; negative affective responses; and risk perception had insignificant direct relations to ERISB.

The second set of hypotheses explored the predictors of information need. Path coefficients for perceived information gathering capacity to information need (β2b = 0.28, *p* < 0.001); informational subjective norms to information need (β2c = 0.37, *p* < 0.001); negative affective responses to information need (β2d = 0.11, *p* < 0.01); and risk perception to information need (β2e = 0.15, *p* < 0.001) were all positive and statistically significant. Surprisingly, the current risk knowledge to information need (β2a = −0.10, n.s.) was negative but non-significant. Hence, H2b, H2c, H2d and H2e were supported and H2a was rejected. These results indicated that informational subjective norms and perceived information gathering capacity had positive impacts on individuals’ information need. Meanwhile, negative affective responses and risk perception were positively related, albeit somewhat weakly, to information need. However, current risk knowledge had no significant effect on information need. 

The third set of hypotheses explored the mediation role of information need. H3a described that information need mediated the positive relationship between current risk knowledge and ERISB. Due to the non-significant association between current risk knowledge and information need (β2a = −0.10, n.s.), H3a was rejected. However, perceived information gathering capacity to information need to ERISB (β3b = 0.08, *p* < 0.001); informational subjective norms to information need to ERISB (β3c = 0.10, *p* < 0.001); negative affective responses to information need to ERISB (β3d = 0.03, *p* < 0.01); and risk perception to information need to ERISB (β3e = 0.04, *p* < 0.001) were all positive and statistically significant, supporting H3b, H3c, H3d and H3e.

The fourth set of hypotheses considered the precursors of current risk knowledge. In this study, both informational subjective norms and negative affective responses had positive influences on current risk knowledge with the standardized coefficients of 0.33 (*p* < 0.001) and 0.39 (*p* < 0.001), in support of H4a and H4b. 

In addition, the other two explorations for the relationships, risk perception to negative affective responses (β5 = 0.39, *p* < 0.001), and current risk knowledge to perceived information gathering capacity (β6 = 0.66, *p* < 0.001). Research found that risk perception had positive influence on negative affective responses, and current risk knowledge had remarkable influence on perceived information gathering capacity, therefore, H5 and H6 were accepted.

## 5. Discussion

The first research objective was to map the direct determinants on earthquake risk information seeking behavior. Figure 3 shows that only information need and informational subjective norms directly affect earthquake risk information seeking behavior. Consistent with former studies [2,3,8,13,16,17,19], an individual who perceived a stronger desire for earthquake risk information was more motivated to seek information. In addition, an individual who felt higher social pressure or expectation from family and friends to be a knowledgeable person on earthquake risk was also more likely to seek related information in more active ways, which is supported by previous studies [3,6,7,8,9,11,13,16,17,19].

In this study, current risk knowledge; perceived information gathering capacity; negative affective responses; and risk perception had non-significant direct influences on the earthquake risk information seeking behavior. However, these direct relationships have been accepted consistently in previous research. There are two possible reasons: one, that compared to past research, some measured items of the questionnaire in this study had been modified. In this study, current risk knowledge was measured through four items and information need was measured through two items using a five-point Likert-type scale instead of the self-reported rating from 0–100 in previous research. Moreover, risk perception was measured by vulnerability and severity through six items, whereas this variable was assessed by probability and severity in previous studies. As the study area was an earthquake stricken area, the probability indicator was inappropriate for this study, therefore the modification of some measured items may have led to biases in the overall conclusions.

The other possible reason for the different results lies in the diverse research contexts. The major research contexts of previous studies were potential risk settings, where a risk-vulnerable area was selected as the study area. In this research, an earthquake stricken area was chosen as the focus of the study where a recent earthquake swarm hit a thousand times within a short period of time. Local governments and other relevant departments had to maximize their efforts to deliver earthquake hazard information by various channels to earthquake victims. The information rich environment made some victims feel that they already had adequate earthquake hazard knowledge, thus leading to less need for information which stopped their earthquake hazard information seeking behavior. In addition, with the frequency of earthquakes, the victims’ attitudes towards the earthquakes changed from tension, worry and anxiety in the early days to numb emotion and less concerned, resulting in the reduction of activity seeking behavior for earthquake information.

Furthermore, the predictors of information need were explored in the model. The direct relationship between perceived information gathering capacity and information need was explored in this study. The result confirmed that this positive link was statistically tenable, according to the research. Consistent with previous studies [3,6,7,8,9,11,13,17], the informational subjective norms had positive impacts on information need. In addition, negative affective responses had a weak relationship with the need of information, which seemed to support the findings of some previous scholars [3,6,7,9,11,13,16,17,19]. Moreover, risk perception related positively, albeit somewhat weakly, to information need, which again, supported former research [14,40]. Only current risk knowledge had a non-significant effect on information need, thus sharing the same conclusion with Ter Huurne et al. [2,13]. This stated that people who perceived being better prepared wanted to gather relevant information, and felt greater social pressure from people around themselves to have more information about earthquakes, may experience a greater need for information. Furthermore, people who possessed bad emotional reactions, such as feeling themselves to be vulnerable earthquake or worrying about severe earthquakes were found to be more eager for earthquake risk information. What was noteworthy was that the current risk knowledge held by individuals did not impact on their need for information, the reasons for which have been alluded to in the above paragraph.

For the second research objective, particular attention was paid to the mediating effect of information need in the earthquake risk information seeking process. It was found that informational subjective norms; perceived information gathering capacity; negative affective responses; and risk perception had direct effects on information need, and information need had direct effect on earthquake risk information seeking behavior, therefore, the conclusion could be drawn that informational subjective norms; perceived information gathering capacity; negative affective responses; and risk perception had indirect effect on the seeking behavior regarding earthquake risk information via information need. This finding seemed to oppose the outcomes of previous studies, where information need (akin to perceived knowledge insufficiency) failed to act as a mediator [6,14]. To our knowledge, this is the first successful effort in verifying the mediating role of information need in a statistical and academic way. 

These were some other outcomes in this study. It was noted that those feeling more negative about the earthquakes would make themselves obtain greater knowledge in order to reduce uncertainty, which is contrary to Ter Huurne et al. finding [13]. In addition, in accordance with prior studies [6,13,16,19], an individual who was conscious of the expectation from relevant others to possess top level risk knowledge about earthquakes, would report themselves to be more knowledgeable. Furthermore, risk perception affected negative affective responses, indicating that negative affect may be strengthened by higher risk perception, again, in line with previous findings [3,6,7,13,16,17]. In this study, we found that current knowledge had a positive impact on the perceived information gathering capacity. Thus, previous knowledge could enhance the gathering capacity to gain new information, in support of the former studies [3,6,8,13]. It was noteworthy that some scholars explored the opposite influence direction, and also found that the relationship was tenable [19,37], which is a subject for future study.

## 6. Conclusions

This article investigated the information seeking behavior of individuals within the setting of earthquake risk in Chinese society, which successfully expanded on the application of the RISP model and related work in earthquake science. Based on the existing models regarding information seeking behavior, the determinants of earthquake risk information seeking behavior, as well as the associations among these predictors were explored. In addition, the role of the individuals’ information need in the seeking process was fully explored in this analysis. Compared to a survey of respondents of a place only potentially vulnerable to a risk, the survey presented to the victims of an earthquake stricken area, provided more realistic and persuasive results, the results of which are enunciated below.

The information need of the victims played a vital role in the earthquake risk information seeking process. Information need was detected as both a direct determinant of earthquake risk information seeking behavior and as a mediator between earthquake risk information seeking behavior and the predictors. Informational subjective norms drove the earthquake risk information seeking behavior in both a direct and indirect ways via information need. Furthermore, perceived information gathering capacity, negative affective responses and risk perception had an indirect effect on earthquake risk information seeking behavior via information need.

In viewing these results, some limitations of this study should be elaborated. (1) Respondents lacked in the elderly and younger social groups, who are also vulnerable during such disasters. However, in this study, the percentage of respondents older than 60 was 3%, and was without any respondents younger than 14 years old. To date, few studies have yet to investigate the risk information seeking behaviors of the elderly and the young, which is an area of interest for future study; (2) Consistent with prior studies, this study targeted the effect of negative affective responses on individual-level information seeking behavior. However, future studies will also examine the influence of positive feelings, such as optimism, on risk information seeking behavior.

Despite these limitations, there are some implications for risk communication theory and practice. Theoretically, based on the RISP model, we improved the measure of the current risk knowledge. The new measured items of current risk knowledge concerned the public’s understanding of what caused earthquakes and how to be ready for them, or responded when they hit. We have also added an additional relationship between perceived information gathering capacity and information need. There are also contributions that can be highlighted for effective risk communication efforts. This research provided a better understanding of the seeking behavior regarding risk information of an individual. Based on it, governments and other stakeholders need to stand ready to improve information strategies that will resonate with their vulnerable audiences. For instance, communicators can (1) highlight the importance of interpersonal communication in information strategies, in everyday living or in times of high risk, since the perceived social pressure (from important others) to be informed about the risk may increase the desire for greater risk information and more active seeking; (2) make audiences perceive their ability to find risk information, manage their emotional response and increase their awareness levels, in order to provoke their risk information need; and (3) use the internet and other information sources to help people acquire information that they are not likely to be in possession of and that they most likely need.

## Figures and Tables

**Table 1:** Overview of exploratory factor analysis and reliability analysis.

Measures	Factor Loadings	Items-Total Correlation	Mean (S.D.)
1. Information seeking behavior (Behavior, α = 0.71)			
In my daily life, when talking about the topic of Songyuan earthquake, I’ll search for as much relative information as possible about this topic (Beha1).	0.77	0.38	3.76 (0.94)
I will search for information about what can I do facing an earthquake (Beha2).	0.74	0.38	4.14 (0.88)
If an earthquake happens in Songyuan, I am likely to search for information (Beha3).	0.77	0.36	3.99 (0.82)
2. Current knowledge (Knowledge, α = 0.77)			
I know a lot about how to avoid disasters, do self-relief and buddy-aid during an earthquake (Know1).	0.70	0.46	3.79 (0.90)
I know a lot about the magnitudes, causes and types of earthquake (Know2).	0.80	0.42	3.39 (0.92)
I know a lot about the earthquakes occurred in local history (Know3).	0.75	0.40	3.11 (0.95)
I know a lot about the evacuation routes and local shelters (Know4).	0.66	0.51	3.12 (1.07)
3. Information need (Need, α = 0.86)			
I need a lot of information to estimate the earthquake risks I am exposed to (Need1).	0.86	0.48	3.86 (0.96)
I need to know everything about the earthquake in my surrounding (Need2).	0.85	0.52	3.90 (1.01)
4. Perceived information gathering capacity (Capacity, α = 0.64)			
If I wanted to get more information about Songyuan earthquake…			
I know what to ask to the experts (Capa1).	0.60	0.47	3.15 (0.99)
I know where to go for more information (Capa2)	0.68	0.45	3.31 (0.93)
I can readily take the time to gather any additional information I need (Capa3).	0.74	0.44	3.10 (1.04)
5. Informational subjective norms (Norm, α = 0.80)			
People who are important to me think that I should stay on top of information about the Songyuan earthquake (Norm1).	0.77	0.48	3.15 (0.97)
I think I should stay on top of information about the Songyuan earthquake (Norm2).	0.84	0.49	3.53 (0.95)
People who I care about also try to collect information about the Songyuan earthquake (Norm3).	0.75	0.49	3.68 (0.90)
6. Negative affective responses (Affect, α = 0.75)			
Songyuan earthquake made me feel tense (Affe1).	0.56	0.55	3.60 (0.91)
Songyuan earthquake made me feel worried (Affe2).	0.87	0.36	3.62 (0.92)
Songyuan earthquake made me feel anxious (Affe3).	0.85	0.40	3.42 (0.97)
7. Risk perception (Perception, α = 0.88)			
Songyuan city is particularly vulnerable to earthquake disasters (Perc1).	0.78	0.51	3.20 (1.19)
In my opinion, the risk of severe earthquake in the future will be greater (Perc2).	0.71	0.52	3.36 (1.10)
If earthquake occurs, I will be directly affected (Perc3).	0.72	0.59	3.74 (1.09)
I perceive an earthquake risk as particularly severe (Perc4).	0.82	0.55	3.58 (1.08)
An extreme earthquake will have negative long-term influence (Perc5).	0.80	0.54	3.38 (1.10)
Earthquake can be catastrophic (Perc6).	0.81	0.55	3.42 (1.08)

Extraction Method: Principal Component Analysis, eigenvalues >1; Rotation Method: Varimax with Kaiser Normalization; Cronbach’s α for scale reliability; Items-total correlation for scale validity; Five-point scale, ranging from 1 to 5 (higher score indicates higher agreement). Beha: behavior; Know: knowledge; Capa: capacity; Affe: affect; Perc: perception. S.D.: Standard Deviation.

**Table 2:** Overall confirmatory factor analysis for the measurement model.

Factor	Measurement Items	Construct Loadings	SMC (R^2^)	CR	AVE
Behavior	Beha1	0.63 (***)	0.40	0.61	0.35
	Beha2	0.56 (***)	0.31		
	Beha3	0.58 (***)	0.34		
Knowledge	Know1	0.57 (***)	0.32	0.75	0.43
	Know2	0.68 (***)	0.46		
	Know3	0.66 (***)	0.43		
	Know4	0.70 (***)	0.49		
Need	Need1	0.81 (***)	0.66	0.85	0.73
	Need2	0.90 (***)	0.81		
Capacity	Capa1	0.66 (***)	0.44	0.64	0.37
	Capa2	0.59 (***)	0.35		
	Capa3	0.57 (***)	0.32		
Norm	Norm1	0.70 (***)	0.49	0.76	0.51
	Norm2	0.78 (***)	0.61		
	Norm3	0.66 (***)	0.44		
Affect	Affe1	0.52 (***)	0.27	0.72	0.47
	Affe2	0.72 (***)	0.52		
	Affe3	0.78 (***)	0.61		
Perception	Perc1	0.87 (***)	0.76	0.93	0.68
	Perc2	0.74 (***)	0.55		
	Perc3	0.78 (***)	0.61		
	Perc4	0.86 (***)	0.74		
	Perc5	0.85 (***)	0.72		
	Perc6	0.85 (***)	0.72		
λ^2^ = 804.34, df = 231, λ^2^/df = 3.48; RMR = 0.05, GFI = 0.93, AGFI = 0.91, PGFI = 0.72, CFI = 0.93, PNFI = 0.76, PCFI = 0.78, RMSEA = 0.05.					

Behavior: Information seeking behavior; Knowledge: Current risk knowledge; Need: Information need; Capacity: Perceived information gathering capacity; Norm: Informational subjective norms; Affect: Negative affective responses; Perception: Risk perception. SMC: Squared multiple correlation; CR: Composite reliability; AVE: Average variance extracted. df: degree of freedom; RMR: root mean square residual; GFI: goodness of fit index; AGFI: adjust goodness of fit index; PGFI: parsimony goodness of fit index; CFI: comparative fit index; PNFI: parsimony-adjusted normed fit index; PCFI: parsimony-adjusted comparative fit index; RMSEA: root mean square error of approximation. Significance level: *** *p* < 0.001.

**Table 3:** The summary of model fit indices.

Fit Index	Absolute Indices				Relative Indices			Parsimony Indices		
	λ^2^/df	GFI	AGFI	RMSEA	NFI	IFI	CFI	PGFI	PNFI	PCFI
Criteria	<5.00	>0.90	>0.90	<0.08	>0.90	>0.90	>0.90	>0.50	>0.50	>0.50
Model	4.42	0.91	0.89	0.06	0.88	0.91	0.91	0.72	0.76	0.78

NFI: normed fit index; IFI: incremental fit index.

**Table 4:** Tests of the hypotheses.

Path	Proposed Direction	Standardized Coefficient	*t*-Value	Result
H1a: Knowledge → Behavior (β1a)	+	0.08	1.14	Rejected
H1b: Need → Behavior (β1b)	+	0.28 (***)	5.68	Accepted
H1c: Capacity → Behavior (β1c)	+	0.10	1.35	Rejected
H1d: Norm → Behavior (β1d)	+	0.30 (***)	6.03	Accepted
H1e: Affect → Behavior (β1e)	−	−0.01	−0.20	Rejected
H1f: Perception → Behavior (β1f)	+	0.01	0.13	Rejected
H2a: Knowledge → Need (β2a)	−	−0.10	−0.14	Rejected
H2b: Capacity → Need (β2b)	+	0.28 (***)	4.16	Accepted
H2c: Norm → Need (β2c)	+	0.37 (***)	8.67	Accepted
H2d: Affect → Need (β2d)	+	0.11 (**)	2.47	Accepted
H2e: Perception → Need (β2e)	+	0.15 (***)	3.84	Accepted
H4a: Affect → Knowledge (β4a)	+	0.39 (***)	8.41	Accepted
H4b: Norm → Knowledge (β4b)	+	0.33 (***)	7.90	Accepted
H5: Perception → Affect (β5)	+	0.39 (***)	8.93	Accepted
H6: Knowledge→ Capacity (β6)	+	0.66 (***)	11.56	Accepted

Behavior: Information seeking behavior; Knowledge: Current risk knowledge; Need: Information need; Capacity: Perceived information gathering capacity; Norm: Informational subjective norms; Affect: Negative affective responses; Perception: Risk perception. Significance level: *** *p* < 0.001, ** *p* < 0.01.
